# Sudden Death With Vasospastic Angina That Could Not Be Medically Managed

**DOI:** 10.7759/cureus.99544

**Published:** 2025-12-18

**Authors:** Murad Emar, Ali Bhatty

**Affiliations:** 1 Department of Cardiology, Hull University Teaching Hospitals NHS Trust, Hull, GBR

**Keywords:** cardiac arrest, coronary artery spasm, coronary vasospastic angina, prinzmetal’s angina, variant angina, vasospastic angina, ventricular pauses

## Abstract

Vasospastic angina is characterized by transient coronary artery spasm leading to myocardial ischemia and may present with ST-segment elevation, arrhythmias, syncope, or cardiac arrest. Although typically responsive to vasodilator therapy, a minority of patients develop medically refractory disease with life-threatening complications. We report the case of a man in his 50s who experienced recurrent episodes of chest pain and syncope over several months, each associated with transient anterior ST-segment elevation and occasional dynamic troponin elevation. Coronary angiography and cardiac MRI demonstrated normal coronary anatomy and no structural heart disease. During admission, telemetry captured a prolonged ventricular pause (~10 seconds) secondary to diltiazem. Despite treatment with calcium-channel blockers, long-acting nitrates, and placement of an implantable loop recorder for rhythm surveillance, he continued to experience intermittent vasospastic episodes. Beta-blocker therapy was withdrawn due to concern for heart block, nitrate therapy was progressively uptitrated in the outpatient setting, and a dihydropyridine calcium-channel blocker was introduced. Several days after his final presentation, with chest pain and normal ECG and biomarkers, he suffered an out-of-hospital cardiac arrest due to ventricular fibrillation and died in the intensive care unit. This case illustrates a rare but severe form of vasospastic angina that remained constrained and refractory to escalating medical therapy and ultimately resulted in a fatal ventricular arrhythmia. Early recognition, optimization of vasodilator therapy, and careful rhythm surveillance are essential, but even with appropriate management, some patients remain at risk for sudden cardiac death.

## Introduction

Vasospastic angina represents myocardial ischemia resulting from transient, inappropriate constriction of one or more epicardial coronary arteries, producing a dynamic reduction in coronary blood flow. Patients who experience angina or ischemia despite non-obstructive coronary arteries often have reduced quality of life and an increased risk of disability, as well as a higher burden of adverse outcomes, including morbidity, mortality, recurrent hospital admissions, repeat angiographic evaluations, and greater healthcare utilization [1]. Unlike classic angina, vasospastic angina is not triggered by exertion. It is characterized by transient ST-segment elevation, often accompanied by reciprocal ST-segment depression during pain episodes. By contrast, typical angina pectoris arises from increased myocardial oxygen demand, is relieved by rest or nitroglycerin, and is associated with ST-segment depression on the ECG during symptomatic periods [2]. We report the case of a patient with recurrent vasospastic angina presenting with classic ST-segment elevation and profound ventricular pauses. This patient’s condition was resistant to standard medical therapy, ultimately leading to death. This case highlights the potentially lethal, challenging, and medically refractory nature of severe vasospastic angina.

## Case presentation

**Figure 1:**
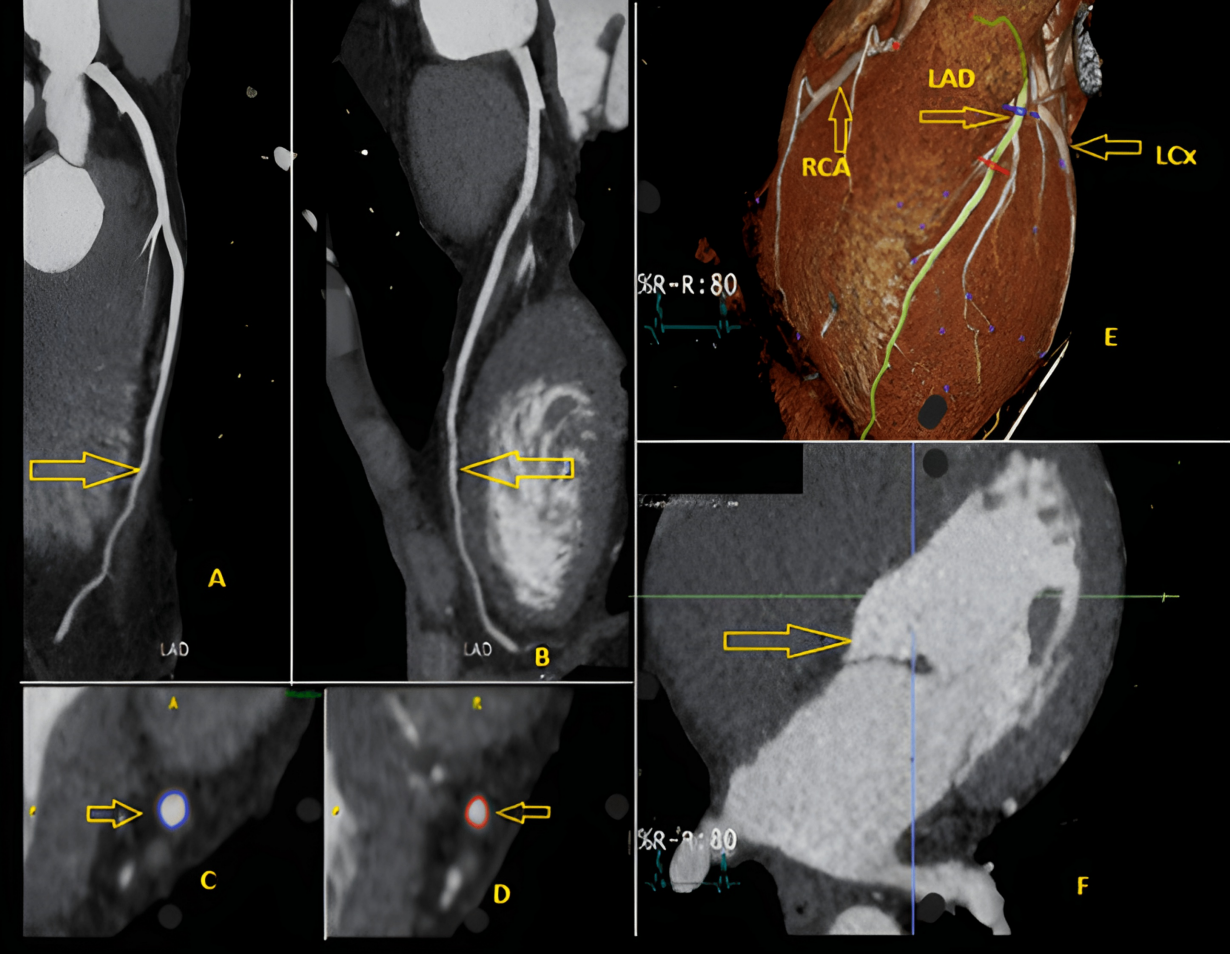
CT coronary angiogram (performed four years prior) showing absence of coronary calcification and a small non-obstructive plaque in the distal left anterior descending artery. (A–D) Curved multiplanar reformations of the left anterior descending (LAD) coronary artery demonstrating a patent vessel with mild, non-obstructive distal atherosclerotic plaque (yellow arrows). No flow-limiting stenosis or high-risk plaque features are identified. (E) Three-dimensional volume-rendered reconstruction showing the coronary tree, including the LAD, right coronary artery (RCA), and left circumflex artery (LCx), all without significant abnormalities. (F) Short-axis view of the left ventricle demonstrating normal cavity size and myocardial wall thickness.

A man in his 50s with a history of asthma and depression presented with recurrent episodes of central, left-arm-radiating chest pain, some associated with transient loss of consciousness. These episodes, initially sporadic, had become more frequent in recent months and occurred without identifiable triggers. Notably, he had been evaluated in a chest pain clinic four years earlier for recurrent chest pain. At that time, the episodes were unexplained and lacked an identifiable precipitant or pattern. At that earlier assessment, a CT coronary angiogram (Figure 1) demonstrated no coronary calcification and only a minor non-obstructive plaque in the distal left anterior descending artery.

**Figure 2:**
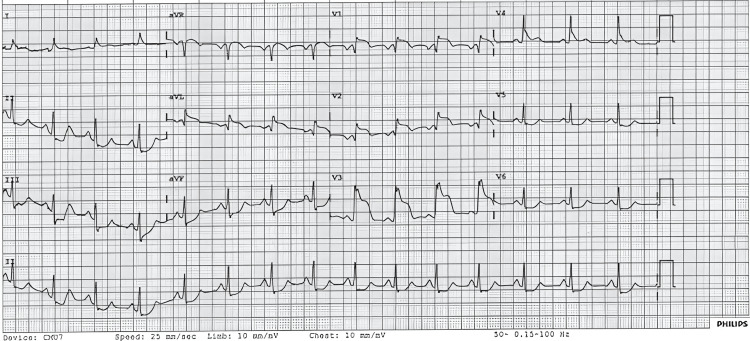
12-lead electrocardiogram obtained during a symptomatic episode demonstrating ST-segment elevation in leads V1–V4 with reciprocal ST depression and T-wave changes in leads V5–V6.

**Figure 3:**
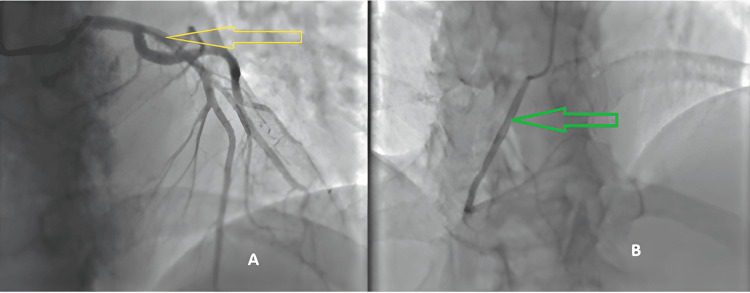
Coronary angiography demonstrating unobstructed coronary arteries without angiographic stenosis. (A) Coronary angiogram in an anteroposterior (AP) cranial projection demonstrating a normal left anterior descending (LAD) artery (yellow arrow). (B) Coronary angiogram in an AP cranial projection demonstrating a normal right coronary artery (RCA) (green arrow).

**Figure 4:**
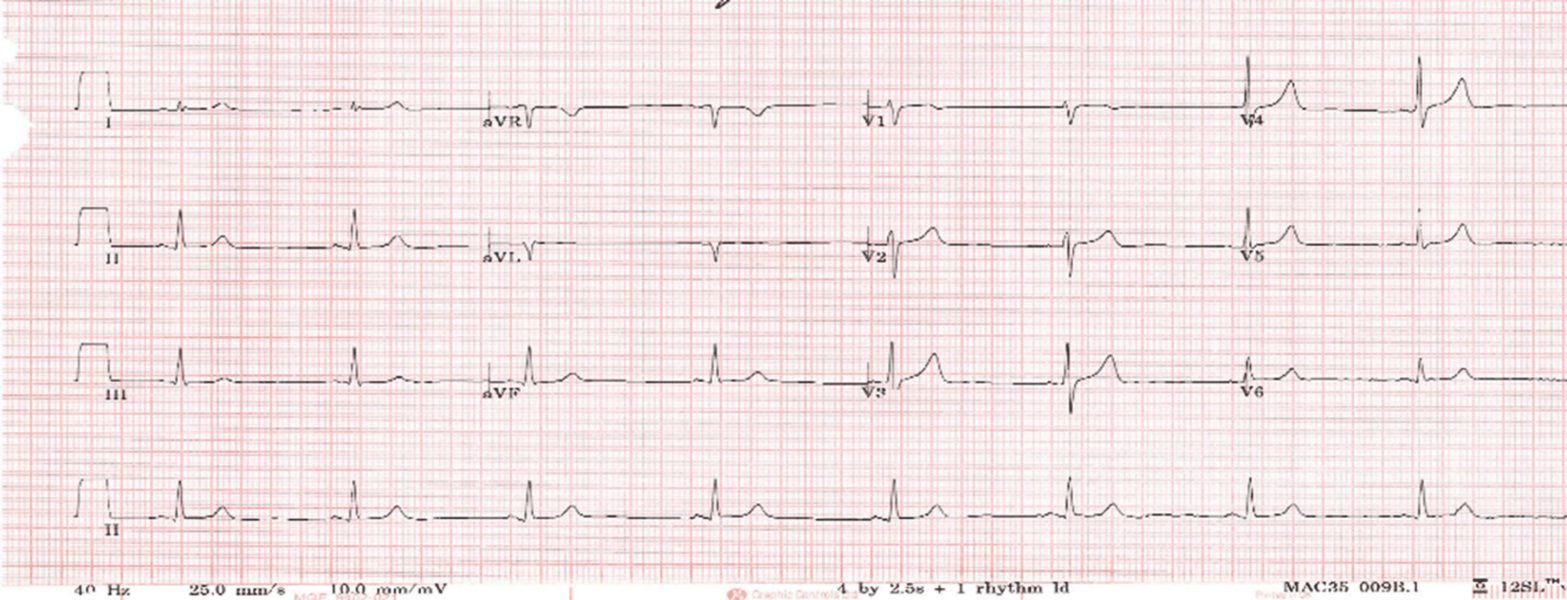
Resolution ECG recorded during admission showing normalization of ST segments following clinical improvement.

Four years later, he began to experience increasingly severe symptoms, prompting multiple admissions to the cardiology department. During one event, the patient experienced syncope lasting approximately two minutes. ECG performed during that episode showed ST-segment elevation in V1-V4 with reciprocal ST depression and T-wave changes in V5-V6 (Figure 2). Cardiac troponin levels were elevated. Coronary angiography revealed normal coronary anatomy (Figure 3), excluding obstructive coronary artery disease. CT pulmonary angiography was negative for pulmonary embolism. This episode was treated as a myocardial infarction with non-obstructive coronary arteries (MINOCA); the patient’s inpatient course was uncomplicated and lasted approximately 48 hours. Transthoracic echocardiography demonstrated preserved left ventricular systolic function, and follow-up ECGs during the admission showed complete resolution of the ischemic changes (Figure 4). He was initiated on beta-blocker therapy and dual antiplatelet therapy and discharged with an arranged outpatient cardiac MRI.

**Figure 5:**
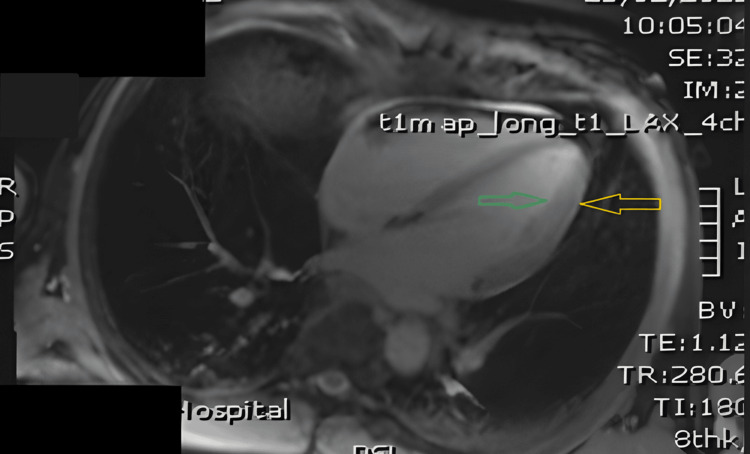
Cardiac MRI demonstrating preserved left ventricular size and systolic function. Long-axis, four-chamber, T1-mapping, cardiac MRI demonstrating normal biventricular size and morphology, with uniform myocardial signal and no evidence of edema or fibrosis. The left ventricular myocardium (yellow arrow) and endocardium (green arrow) appear normal. No pericardial effusion is present, and the atria are normal in size with no structural abnormalities identified.

**Figure 6:**
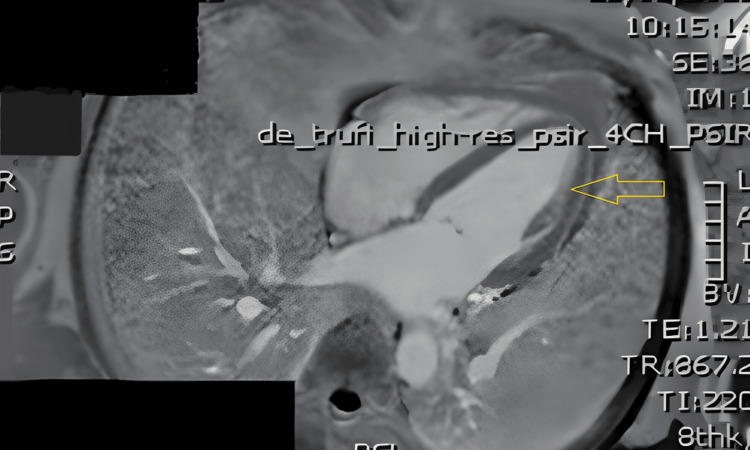
Cardiac MRI showing no evidence of myocardial scar or focal fibrosis. TrueFISP (TRUFT) cine cardiac MRI sequence demonstrating normal cardiac structure and function, with a normal-appearing left ventricular (LV) myocardium (yellow arrow).

Several weeks later, he was readmitted after a second collapse and remained an inpatient for seven days. The episode occurred while cycling; he developed chest pain, used sublingual glyceryl trinitrate, and then lost consciousness for about 15 minutes. While hospitalized, the patient experienced additional episodes with similar ECG changes. Telemetry captured a prolonged ventricular pause (~10 seconds) with bradycardia. Cardiac MRI results were available then and showed normal ventricular structure and function with no evidence of myocarditis, scarring, or infiltrative disease (Figures 5, 6). During this admission, he was initially reintroduced to beta-blocker therapy and subsequently commenced on diltiazem, after which he developed clinically significant drug-induced atrioventricular block. He continued to have recurrent episodes of resting chest pain with transient ST-segment elevations that subsequently resolved. No sustained ventricular arrhythmia was recorded on telemetry during the seven-day stay. A multidisciplinary electrophysiology team reviewed the case and concluded that the overall presentation was most consistent with vasospastic angina complicated by bradyarrhythmia. The electrophysiology multidisciplinary team concluded there was no current indication for permanent pacing or for an implantable cardioverter-defibrillator (ICD). An implantable loop recorder was placed for continuous rhythm surveillance. He was discharged on a calcium-channel blocker (amlodipine), oral nitrates, and a low maintenance dose of bisoprolol (1.25 mg).

**Figure 7:**
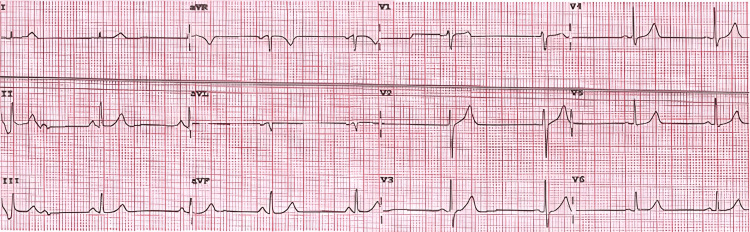
12-lead ECG recorded at the final clinic visit, demonstrating normal conduction intervals and no acute ischemic changes.

Two weeks later, he attended same-day emergency care with recurrent chest pain; serial troponin measurements were within normal limits (10 ng/L), and the ECG obtained during assessment was unremarkable, so he was reassured and discharged. Over the following two months, he attended two cardiology outpatient reviews. At the first visit, bisoprolol was discontinued, and he remained on amlodipine 5 mg and isosorbide mononitrate 30 mg modified release once daily. Sertraline was commenced to address a significant deterioration in his mental health and work-related inactivity. He reported improved exercise tolerance, cycling up to 10 miles, with no further syncopal episodes; intermittent chest discomfort persisted, predominantly on awakening. At the second clinic visit, he described ongoing intermittent morning chest pain relieved by glyceryl trinitrate spray but otherwise tolerated increasing physical activity. His isosorbide mononitrate dose was uptitrated to 60 mg modified release once daily. One week after the last clinic review, he again attended the same-day emergency care with recurrent chest pain; troponin levels and ECG (Figure 7) were both normal. At that time, his regimen included amlodipine 7.5 mg and isosorbide mononitrate 60 mg. This proved to be his final clinical assessment before his presentation with an out-of-hospital cardiac arrest several days later. Throughout all admissions and outpatient reviews, he showed no evidence of left ventricular failure and maintained stable blood pressure recordings. Apart from intermittent pauses, no clinically significant arrhythmias were observed on telemetry. The QTc and other ECG intervals remained within normal limits. The ILR did not register any actionable events and was not interrogated in the hours preceding his final collapse. During the final presentation, paramedics were called to his home; the on-scene ECG documented ventricular fibrillation. He was transferred to the intensive care unit but died later the same day, leaving behind a bereaved spouse and family. He had presented to a hospital on nearly eight occasions over a six-month period before his final out-of-hospital cardiac arrest, which was documented as ventricular fibrillation.

## Discussion

Coronary artery spasms in vasospastic angina most commonly occur at rest, particularly between midnight and early morning, when arterial tone is elevated and goes through “hot” and “cold” phases. Although daytime exertion rarely provokes them, spasms can be triggered by various stimuli or medications, especially in the morning. Calcium-channel blockers such as diltiazem and nifedipine are highly effective in preventing these episodes [3].

Pathophysiologically, vasospastic angina is marked by abnormal vasomotion, or irregular rhythmic contraction, of one or more coronary arteries [4]. Clinically, these episodes often present as chest pain at rest, accompanied by transient ECG changes such as ST-segment elevation. The condition may mimic angina or even myocardial infarction, despite the absence of significant coronary artery obstruction [5].

Diagnosis is based on a combination of clinical presentation, ECG findings, and evidence of coronary artery spasm. A key feature is angina that responds to nitrates, typically occurring at rest, most often during nighttime or early morning. Patients may also experience reduced morning exercise tolerance, hyperventilation-triggered episodes, and improvement with calcium-channel blockers, while beta-blockers are generally ineffective. Diagnostic confirmation involves detecting transient ischemic changes on the ECG during an episode, such as ST-segment elevation or depression of ≥0.1 mV, or new negative U waves in at least two contiguous leads. Coronary spasm is identified as a temporary narrowing greater than 90% that induces both chest pain and ischemic ECG changes, which may occur spontaneously or be provoked using agents such as acetylcholine, ergot derivatives, or hyperventilation [6].

In emergency settings, management of vasospastic angina follows established acute coronary syndrome protocols, with treatment tailored to the patient’s presenting features. This ensures timely recognition and appropriate intervention according to the severity and characteristics of the symptoms observed at presentation [7].

Calcium-channel blockers remain the cornerstone of long-term management, reducing spasm frequency and improving outcomes [8]. Long-acting nitrates, which act through a complementary mechanism, can be used alongside calcium-channel blockers to enhance symptom control. Nicorandil has also been shown to lower the burden of anginal episodes and improve symptom relief [9-11].

A large Finnish cohort study of 1,762 hospital admissions for vasospastic angina reported a cardiac mortality rate of 11.1%, with a higher risk observed in patients without angiographically detectable coronary obstruction [12]. The role of ICDs in vasospastic angina remains insufficiently defined in both the literature and current guidelines. Nonetheless, in patients considered at high risk for ventricular arrhythmias, ICD implantation may be used as a secondary prevention measure to reduce morbidity and mortality [13]. Despite this, cases of cardiac arrest have been documented in individuals with vasospastic angina who were receiving optimal medical therapy and had an ICD in situ [14].

## Conclusions

This case underscores that vasospastic angina, although often responsive to vasodilator therapy, can follow a relentlessly recurrent and ultimately fatal course despite apparently appropriate medical management and close surveillance. Recurrent transient ST elevation, dynamic biomarker changes, prolonged ventricular pauses, and multiple hospital presentations in the absence of obstructive coronary disease should alert clinicians to a high-risk phenotype in which standard therapy (calcium-channel blockers and nitrates) may be insufficient. The case further illustrates limitations of current risk-stratification strategies and monitoring modalities, including implantable loop recorders, in predicting catastrophic ventricular arrhythmia. Management of such patients requires a multidisciplinary approach that balances aggressive spasm suppression (optimizing calcium-channel blockers, nitrates, and consideration of alternative agents such as nicorandil) with careful electrophysiological assessment and individualized discussion of device therapy where appropriate. Given the absence of robust guideline-directed criteria for ICD implantation in vasospastic angina, clinicians must assess risk on a case-by-case basis and consider earlier referral to specialist electrophysiology services for high-risk patients. Finally, this report highlights an urgent need for prospective data to define predictors of malignant arrhythmic events in vasospastic angina and to guide decisions about advanced therapies aimed at preventing sudden cardiac death.
